# Biological evaluation of new potential anticancer agent for tumour imaging and radiotherapy by two methods: ^99m^Tc-radiolabelling and EPR spectroscopy

**DOI:** 10.1080/13102818.2014.978666

**Published:** 2014-11-25

**Authors:** Yanka Karamalakova, Krishna Chuttani, Rakesh Sharma, Antoaneta Zheleva, Veselina Gadjeva, Anil Mishra

**Affiliations:** ^a^ Department of Chemistry and Biochemistry, Medical Faculty, Trakia University, 11 Armeiska Street, 6000Stara Zagora, Bulgaria; ^b^ Division of Cyclotron and Radiopharmaceutical Sciences, Institute of Nuclear Medicine and Allied Sciences, Brig S.K. Mazumdar Marg, Delhi110054, India; ^c^ Institute of Nuclear Medicine and Allied Sciences, Brig S.K. Mazumdar Marg, Delhi110054, India

**Keywords:** SLENU, *ex vivo* EPR, ^99m^Tc-conjugate, biodistribution, *EAT tumour*, tumour imaging

## Abstract

Recently, a new class of in vitro and ex vivo radiotracers/radioprotectors, the nitroxyl-labelled agent 1-ethyl-1-nitroso–3-[4-(2,2,6,6–tetramethylpiperidine-1-oxyl)]-urea (SLENU), has been discovered. Our previous investigations demonstrated that SLENU is a low-molecular-weight stable free radical which is freely membrane permeable, easily crosses the blood brain barrier and exhibited in/ex vivo the lowest general toxicity and higher anticancer activity against some experimental tumour models. Further investigation was aimed to develop a ^99m^Tc-labelled SLENU (97%) as a chelator and evaluate its labelling efficiency and potential use as a tumour seeking agent and for early diagnosis. Tissue biodistribution of ^99m^Tc-SLENU was determined in normal mice at 1, 2 and 24 h (*n* = 4/time interval, route of administration i.v.). The distribution data were compared using male albino non-inbred mice and electron paramagnetic resonance investigation. The imaging characteristics of ^99m^Tc-SLENU conjugate examined in BALB/c mice grafted with *Ehrlich Ascitis tumour* in the thigh of hind leg demonstrated major accumulation of the radiotracer in the organs and tumour. Planar images and auto-radiograms confirmed that the tumours could be visualized clearly with ^99m^Tc-SLENU. Blood kinetic study of radio-conjugate showed a bi-exponential pattern, as well as quick reduced duration in the blood circulation. This study establishes nitroxyls as a general class of new spin-labelled diagnostic markers that reduce the negative lateral effects of radiotherapy and drug damages, and are appropriate for tumour-localization.

## Introduction

Modern chemotherapy along with surgery and radiation therapy is still the most efficient methods of cancer treatment. The increased production of reactive oxygen species (ROS) however could be a reason for many dangerous side effects that sometimes hamper the therapy and may lead to serious or even fatal organ dysfunctions. Strategies to attenuate drugs and radiation toxicity include dosage optimization, synthesis and the use of analogues having lower toxicity or a combined therapy with antioxidants. Clinical and experimental trials have been directed toward development of new antioxidants as anticancer agents to be applied individually or conjugated with toxins, drugs, natural extracts and antitumour radio-isotopes for chemotherapeutic and radiotherapeutic treatments.[1–3] Isotope radiolabelling (^99m^Tc-labelled) of active anticancer agents has been also introduced for attaching the radio-isotope to the antitumour drugs in order to increase the effectiveness of interactions at cell level.[4,5] Currently, the radiopharmaceuticals combined with direct radiography are the only options for investigation of the location of tumour malformations and their visualization by gamma scintigraphic (real time) imaging of the body.[6–8]

The clinically used antitumour drug Lomustine 1-(2-chloroethyl)-3-cyclohexyl-1-nitrosourea (CCNU) indicated higher clinical activity against human malignancies, variety of human neoplasms, lymphomas, melanomas, some solid tumours, testicular carcinomas, Hodgkin's disease and brain tumours. Simultaneously with improvement of the health-related quality of the patients, this 2-chloro nitrosourea exhibits high toxicity against the normal cells, responsible for distortions to the integrity of the subsequent DNA and induced chromosomal aberration.[9,10] Various types of drug formulations have been used as drug delivery vehicles for sustained release of proteins and peptides and some have been evaluated for clinical applications. These formulations have been used as carriers of cytotoxic drugs with the strategy based on reduction of toxicity and passive delivery to tumours.[10–12] These facts are a prerequisite for the synthesis and research of new derivate of Lomustine.

Reduced toxicity and increased antineoplastic properties were achieved when nitroxyl(aminoxyl) groups were introduced in the chemical structure of certain antitumour drugs.[13,14] This finding encourages us to synthesize a number of spin labelled analogues of the anticancer drug CCNU. Some of these spin-labelled nitrosoureas showed advantages over CCNU, having lower toxicity and higher anticancer activity against some experimental tumour models.[15] By electron paramagnetic resonance (EPR) method we have shown that spin-labelled nitrosoureas and their precursor 4-amino TMPO can scavenge. O^2−^ and so exhibit high superoxide scavenging activity (SSA).[16]

**Figure 1.**
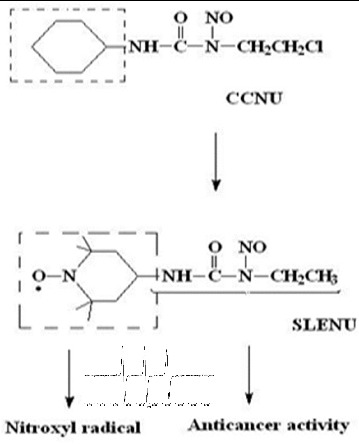
Chemical structures of non-labelled drug CCNU, nitroxyl-labelled analogue SLENU and EPR spectrum of the spin-labelled agent.

1-ethyl-1-nitroso-3-[4-(2,2,6,6-tetramethylpiperidine-1-oxyl)]-urea (SLENU) (Figure 1), recently synthesized in our laboratory, is a spin-labelled analogue of CCNU that migrates readily through the cellular membrane. Our formerly reported results for alkylating, carbamoylating activities and half-life times of SLENU showed that the alkylating activity was lower than that of CCNU, but the carbamoylating activity of SLENU was high and comparable with that of CCNU. The half-lives were also comparable (75 min for SLENU and 54 min for CCNU).[10] Moreover, by our studies, we have demonstrated the beneficial effects of SLENU on drug-induced oxidative toxicity in rat blood and in mice organs.[17,18] Nitroxyl radicals possess high-T_1_ contrast properties and could be used in MRI and EPR imaging investigations. All these characteristics of nitroxyl radical in SLENU, and all applications in life science research make the spin-labelled drug attractive for MRI and EPR organ diagnostics, and for further investigations with high susceptible ex vivo techniques.

The conventional method for investigation of paramagnetic (spin-labelled) agents, their organ distribution, including the ex vivo tissue homogenates and in vivo live animals is the EPR spectroscopy. This technique provides unique details that allow us to measure and represent processes of the metabolism of free radicals, the ROS, organ/tissue oxygenation and nitrous oxide production (RNS) in the normal physiology and cancer processes. Unfortunately, with EPR/ EPRI spectroscopy the precise organ-specific location of the tumour in the body cannot be determined.[19,20,21] A thorough examination and understanding of the targeting, the visualization and biodistribution in different organs of the spin-labelled compounds could be achieved by radiolabelling with technetium-99m (^99m^Tc).[1–3] ^99m^Tc is the radionuclide of choice in the development of diagnostic imaging agents by virtue of its wide availability, convenient half-life and ideal imaging energy.[6–8,22,23]

Therefore, the aim of the present study was to determine and compare the pharmacokinetic/ biodistribution of the spin-labelled nitrosourea SLENU before and after its labelling with ^99m^Tc. The solubility, the pharmacodynamics of elimination through blood circulation for a short/long time and toxicity of SLENU were also investigated by EPR distribution in organs of normal mice. In the present study, we also demonstrate the accumulation and specific tumour uptake of radioactive conjugate in solid *Ehrlich Ascites Tumour*, toxicity and permeability for BBB after gamma imaging studies.

## Material and methods

### Chemicals

Spin-labelled drug SLENU was previously synthesized by [10]. Stannous chloride dehydrated (SnCl_2_.2H_2_O), the spin-trapping agent, phosphate buffered saline (PBS) and K_3_[Fe(CN_6_)] were purchased from Sigma-Aldrich Chemical Co, St. Louis, USA. ^99m^Tc was procured from Regional Centre for Radiopharmaceuticals, Board of Radiation and Isotope Technology (BRIT), Department of Atomic Energy, India. All other chemicals and solvents were of analytical reagent grade.

### ^99m^Tc-labelling study

SLENU (4 mg) was dissolved in 1 mL distilled water and was labelled with ^99m^Tc by a direct labelling method.[24] Briefly, 0.1 mL stannous chloride solution (1 mg/mL) was added to it and pH was adjusted to 7.0 using 1 mol/L sodium bicarbonate sol. Freshly eluted 2 mCi(74 MBq) ^99m^Tc-pertecnetate in 0.5 mL saline was added, mixed thoroughly and the reaction mixture was incubated for 10–20 min at room temperature (22 °C). After incubation of the radio-conjugate, the radiochemical purity and *in vitro* studies (up to 24 h) were carried out by paper chromatography using ITLC-SG (instant thin layer chromatography-silica gel) paper as the stationary phase, acetone and saline as the mobile phases.

### Instrumentation

HPLC analyses were performed on a Waters chromatograph efficient with 600 coupled to a Waters 2487 photodiode array ultraviolet detector. ITLS-SG (Gelman Sciences Inc., Ann Arbor, MI) was used for labelling efficiency determination. Gamma imaging and biodistribution studies were done using a planner gamma camera (Hawkeye, Germany) and gamma-scintillation counter (Capintec, USA), respectively. All EPR measurements were performed on X-band EPR, spectrometer (Bruker, Germany), equipped with standard Resonator, Bulgaria. Experiments were carried out in triplicate. The EPR spectra were measured at room temperature (300 K) at modulation amplitude 10.00 G and microwave power 1.28 mW.

### Stability study of the ^99m^Tc-SLENU

The percentage labelling efficiency and stability of ^99m^Tc-SLENU at a particular point was performed as per the method described earlier.[4] It was estimated by Ascending ITLC (Gelman Sciences Inc., Ann Arbor, MI) using acetone and pyridine, acetic acid and distilled water (PAW) (3:5:1,5 v/v) as mobile phases, and silica gel (SG)-coated fibre glass strips as the stationary phase. Approximately 2–3 μL of the radio-labelled complex was applied at a point 1 cm from one end of an ITLC-SG strip. The strip was developed in acetone or 0.9% saline and the solvent front was allowed to reach 8 cm from the point of application. The strip was cut horizontally into two halves, and the radioactivity in each segment was determined in a well-type gamma-ray counter calibrated for ^99m^Tc energy. The free ^99m^Tc-pertechnetate that moved with the solvent (*R_f_* = 0.9–1.0) was determined. The reduced/hydrolysed (R/H) technetium remained at the point of application, whereas free pertechnetate and labelled complex moved with the solvent front in PAW.

### Blood kinetics

For EPR experiments of SLENU (administrated i.p. at a dose of 40 mg/kg) blood samples were taken from the free-streaming blood of a male albino non-inbred mice and were collected into heparinized tubes containing PBS (pH = 7–7.4). Blood clearance of ^99m^Tc-SLENU was determined in rabbit by administering intravenously 18.5 MBq of the radio-labelled complex into the dorsal ear vein and thereafter collecting blood samples from the other ear veins of the rabbit starting from 15 min to 24 h post-injection and then counting the samples in the gamma counter. Decay-corrected radioactivity in the blood was expressed as per cent injected dose in the blood, using the total blood volume as 7% of the body weight. Animal protocols have been approved by the Bulgarian/Indian Institutional Animals ethics Committee.

### Tumour line

Experimentally, monolayer cultures for cell experiments of murine cell line EAT (*Ehrlich Ascitis tumour*) were used. EAT was maintained in the peritoneum of the mice in the ascites form by serial weekly passage. The exponentially growing cells were washed and suspended in PBS pH = 7.4. Approximately, 10–15 million cells were subcutaneously injected into the thigh of the right hind leg of the mice (8–12 weeks old, weighing about 25–30 gm). Tumours were allowed to grow for 7–10 days to reach a diameter of approximately 0.9–1 cm and thereafter used for further studies. An injected dose of 120 μCi (100 μL) of ^99m^Tc-SLENU was used.

### EPR organ and blood distribution

First biodistribution study for EPR experiments of SLENU in organ homogenates (liver, lungs, spleen, pancreas, brain, kidneys) and blood was evaluated in male albino non-inbred mice (35–40 g body weight, normal diet) as previously described by [10]. Spin-labelled nitrosourea was administrated i.p. at a dose of 40 mg/kg. Animals were decapitated at appropriate time points following injection (10, 30, 60, 90 min and on 4 h, 24 h) and dissected. Tissues from lungs, liver, spleen, brain, kidneys, pancreas and blood were collected and processed immediately. For nitrosourea extraction, samples were weighed and homogenized in PBS (10% w/v) and centrifuged at 2000 g for 15 min. Supernatants were collected and the concentration of nitrosourea was evaluated by EPR spectroscopy. Before measuring the spin-labelled concentration, the samples were deoxidized by K_3_[Fe(CN_6_)] (a spectroscopic broadening reagent), because of the fast reduction of the nitroxide function (10–20 min) in the tissues.

### ^99m^Tc-biodistribution

The study was performed to assess the distribution and localization of ^99m^Tc-labelled SLENU. An intravenous injection of ^99m^Tc-SLENU in a volume of 100 μL was injected through the tail vein of each mouse (Balb/c mice, 22–30 g. body weight, and normal diet). At 1, 4 and 24 h after injection, mice were sacrificed and dissected. Tissues from different organs (liver, kidneys, lungs, muscle, spleen, brain, heart) were removed, cleaned from adhering tissues, weighed and then their radioactivity was measured in a shielded well-type gamma scintillation counter calibrated for ^99m^Tc-energy. Uptake of the radiotracer in each tissue was calculated and expressed as per cent injected dose (activity) per gram of the tissue (% ID/g).

### Gamma scintigraphic imaging in EAT bearing mice

Image viewing was performed using planar gamma camera (Hawkeye).[25] Localization of radio-labelled agent over time is performed in EAT (10-15 million) cells implanted tumour bearing mice by injecting 100 μci ^99m^Tc-SLENU in the tail vein. Images were obtained at different time intervals starting from 1, 2, 4 and 24 h post-injection. Data presented are results from experiments performed in quadruplicate (or three mice in each four groups). Ellipsoid regions of interest (ROIs) based on the gamma images were drawn on the heart, lungs, stomach, muscle, liver, spleen and brain, around the kidneys and the total body. The ROI was also drawn on the contralateral muscle of the mice in the left hind limb. For delineation of the tumour, at threshold of at least 25% of the maximum pixel value was chosen. For the calculation of tumour uptake at 2.5 h after injection, this threshold value was individually adjusted to obtain the same ROI volume as the tumour ROI at 1 h for the same animal. Uptake was calculated as the counts in the tissue divided by the injected activity and normalized for the ROI size (%ID/g). Tracer elimination at 1 and 24 h was calculated by subtracting the total body counts at the time of imaging from the injected activity and expressed as percentage by multiplying with 100 and dividing by the injected activity.

### Statistical analysis

Statistical analysis was performed with Statistica 6.1, Sta-Soft, Inc. and results were expressed as mean ± standard error (SE) or standard deviation (SD). Statistical significance was determined by the Student's *t*-test. A value of *p* < 0.05 was considered statistically significant.

## Results and discussion

SLENU, a nitroxyl free radical analogues of Lomustine, was synthesized and over the last 25 years its properties and activities have been investigated.[9–12,21,26,27]

The structure of spin-labelled agent SLENU analogue of the clinically used drug Lomustine is shown in Figure 1.

The nitrosourea (^1^H-NMR; IR spectra cm^−1^: 1340, 1730, 1540, 1340; anal. C_12_H_23_N_4_O_3,_ 271.12) exhibits powerful SSA.[16] The result for τ_0.5_ (75 min) high carbamoylating activity (54.46 ± 0.17/ EPR research) and low-alkylating activity (0.008 A_560_/mmol/L/h) [11] exhibited the best combination for a good therapeutic index, high antitumour activity and low cytotoxicity. The EPR spectra were symmetric triplet constant strong signals with *a_N_* = 16–17 G, characterization for nitroxyl-labelled six-membered cyclic (nitroxyl) radicals (Figure 1). The beneficial effects are likely attributed to the antioxidant effect of the incorporated nitroxide, and SLENU could be used like potential synergistics of anticancer antibiotics [10,12,15] and protector against the oxidative toxic effects of antitumour antibiotics.[17,18]

Radio-labelled ^99m^Tc-SLENU, the spin-labelled conjugate, was challenged to test the stability, and the labelling efficiency and results are presented in Table 1.

**Table 1.** *In vitro* stability of ^99m^Tc-labelled SLENU conjugate at different time intervals.

Time (h)	% labelled complex	% free TcO_4_	% reduced hydrolysed
0	97%	1.5%	1–1.5%
1	97%	1.5%	1–1.5%
4	97%	1.5%	1–1.5%
24	97%	1.5%	1–1.5%

Optimum conditions required for maximum labelling efficiency were established. The drug was found chemically pure (98.9%) and in vivo stable. It was labelled with ^99m^Tc with more than 97% labelling efficiency and only 1-1.5% degradation was observed for 24 h. Stability of the labelled complex with time was studied in saline at standard conditions (37°C, pH = 7), as shown in Table 1. The high stability of labelled radioactive product for a long time ensures continuing suitability for in/ex vivo use. This spin-labelled agent has high metal stability, rapid organ allocation and uncompromised reactivity.

**Figure 2.**
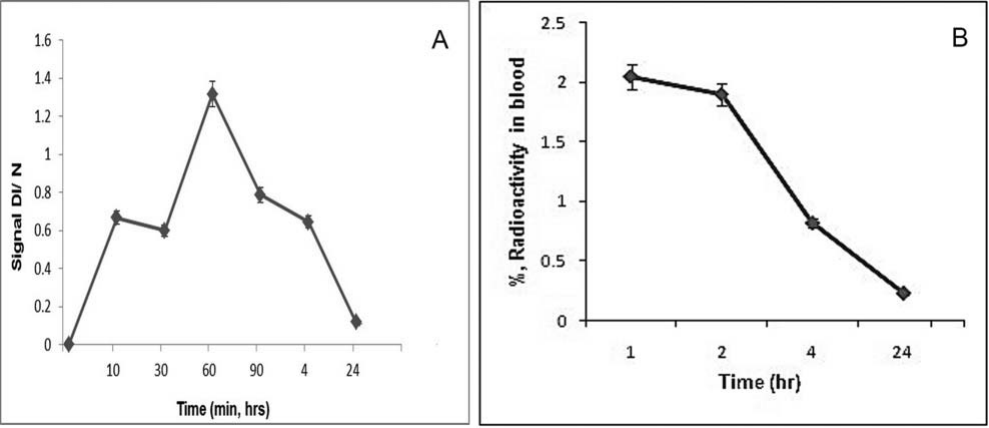
(A) Blood kinetic curve of SLENU of male albino non-inbred mice after i.p. administration. EPR measurements were performed on four groups of three animals. The SE associated with 6% of the presented values. (B) Blood kinetic curve of ^99m^Tc-SLENU of BALB/c mice after i.v. administration. Data from the groups of five mice are expressed as a mean% ID/g ± SD.

Simeonova et al. [28] found that polybutylcyanoacrylate nanoparticles loaded with spin-labelled nitrosourea were localized in the lungs and blood of Lewis lung carcinoma-bearing mice.[28] Based on this finding, the blood kinetics data of the spin-labelled nitrosourea, registered by EPR (Figure 2(A)) and ^99m^Tc-SLENU in blood of healthy mice after labelling with radio-isotope (Figure 2(B)), were investigated, respectively.

The blood samples were collected using a micro-capillary at 10, 30, 60, 90 min and 4 and 24 h p.i. of SLENU and 1, 2, 4 and 24 h p.i. with ^99m^Tc-SLENU. The blood clearance studies of free drug conducted showed that the half-life of the drug was greater than the drug in its free state. The maximum concentration of SLENU, registered by EPR (arbt. units), in blood reached at 60 min after i.p. injection and almost completely observed to 24 h. The maximum concentration of ^99m^Tc-conjugate was reached at 60 min p.i. and then gradually declined.

**Figure 3.**
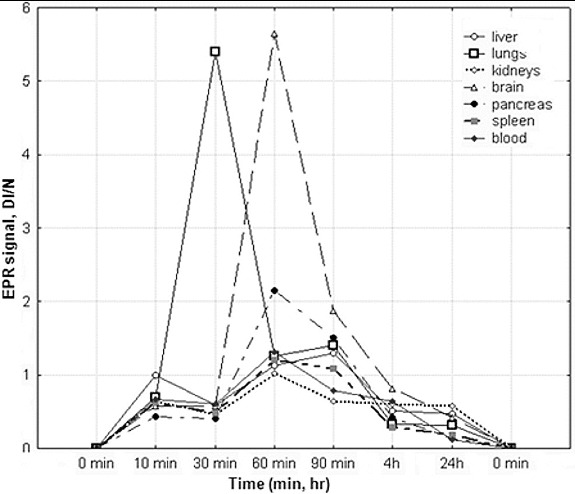
Tissue distribution of SLENU in organ homogenates (liver, spleen, lung, kidneys, pancreas, brain) and blood of albino non-inbred mice after i.p. administration. EPR measurements were performed on four groups of mice/ three animals. The SE associated with 6% of the presented values.

EPR biodistribution of SLENU was investigated in organ homogenates (lungs, liver, spleen, pancreas, brain, kidneys) of male albino non-inbred mice (Figure 3).

The data showed a complete absence of the spin-labelled nitrosourea within 24 h in all tissues studied. In kidneys, brain, pancreas and spleen, the maximum concentration of nitrosourea was seen 60 min after administration of the drug. Only in the liver and lungs, the maximum concentration of nitrosourea was reached on the 90 and 30 min after injection. A relatively low accumulation found in the liver, kidneys and pancreas was a prerequisite for a low toxicity in these organs. The spin-labelled nitrosourea was mainly localized in the lungs and in the brain on the 60 min. The high concentration of the spin-labelled nitrosourea in brain indicates that the compound may be a consequence of the use in treatment of brain tumours. The lower alkylating activity, high carbamoylating activity and half-life time established previously are in agreement with the lower toxicity of the compound on the studied organs. Thus, the low organ/tissue toxicity of the nitroxyl-labelled nitrosourea was attributed to the high antioxidant effect.[10,12, 16] Other authors have also demonstrated the selection of nitroxyl-labelled derivates for in vivo EPR imaging [26,29] and confirmed higher EPR signal intensity of tissue homogenates than the same signal from the TEMPOL-treated animals.[10,21,27,30] Although nitroxyls have a lower relaxivity than conventional contrast agents such as gadolinium and technetium complexes, the volume distribution of nitroxyls is greater because of the better cell permeability.[21, 27, 30]

Based on the above-mentioned facts, we have made the following assumptions to explain the ex vivo maximum labelling efficiency and low-toxicity effect of the ^99m^Tc-SLENU conjugate; and to verify the possible use as a contrast marker for early detection/screening of body-tumour formation and to confirm the tumour localization in the brain tissue.

To evaluate the potential significance of SLENU uptake/elimination by various tissues with regard to cytotoxicity, we monitored the biodistribution of the drug labelled with ^99m^Tc. (Table 2).

**Table 2.** *In vitro* biodistribution study of ^99m^Tc-SLENU in whole body organ homogenates and blood of BALB/c health mice after i.v. administration. Data from the groups of five mice are expressed as a mean% ID/g ± SD at different time intervals.

Organs	% ID/g 1 h	% ID/g 4 h	% ID/g 24 h
Blood	2.05 ± 0.68	0.82 ± 0.26	0.23 ± 0.05
Heart	0.42 ± 0.13	0.11 ± 0.03	0.04 ± 0.01
Lungs	3.7 ± 0.72	1.8 ± 0.31	0.41 ± 0.12
Liver	43.6 ± 4.22	29.19 ± 2.61	9.52 ± 1.04
Spleen	12.9 ± 1.44	6.6 ± 1.37	1.21 ± 0.42
Kidney	3.08 ± 0.81	1.75 ± 0.58	0.88 ± 0.21
Stomach	0.73 ± 0.25	0.41 ± 0.11	0.26 ± 0.08
Intestine	0.92 ± 20.24	0.46 ± 0.14	0.31 ± 0.12
Muscle	0.53 ± 0.16	0.43 ± 0.14	0.21 ± 0.07
Brain	0.12 ± 0.001	0.07 ± 0.001	0.00 ± 0.00

The data demonstrated that the major accumulation of the radio-conjugate (activity in terms of per cent injected dose per gm organ/tissue) was in heart, lung, liver, spleen, kidney, stomach and intestine.

**Figure 4.**
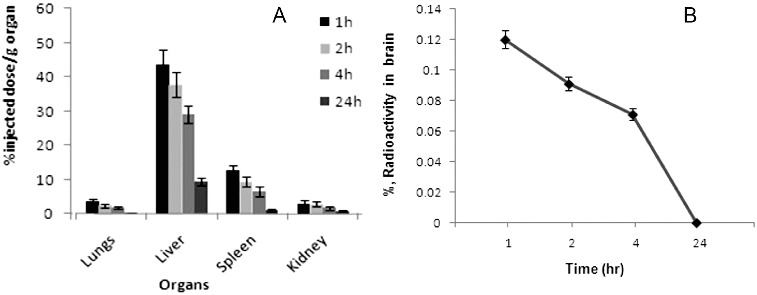
(A) Tissue biodistribution of ^99m^Tc-SLENU in organ homogenates (liver, spleen, lung, kidneys) and (B) in brain homogenates of BALB/c mice after i.v. administration. Data from the groups of five mice are expressed as a mean% ID/g ± SD.

The experimental data revealed that at a period of 24 h incubation, more than 97% binding with ^99m^Tc signifies not only the high stability of the ^99m^Tc-labelled product but also its suitability for in vivo use. The gradual decline of the labelled compound from the circulation suggests its high-protein binding with the plasma proteins. Distribution data demonstrated that the maximum accumulation of radio-conjugate was in liver (43.6 ± 4.22%) followed by spleen (12.9 ± 1.44%), lungs (3.70 ± 0.72%) and kidneys (3.08 ± 0.81%) at 1 h post-injection (Figure 4(A)).

The accumulation of the radio-conjugate in the liver hepatocytes (43.6% /g) at 1 h post-injection shows that the major portion of the conjugate is excreted through the hepatobiliary route. The hepatobiliary clearance was also observed as liver shows decrease (9.52%/g) of radioactivity at 24 h. This may be therapeutically acceptable, since the liver is the usual metabolizing organ for most of the drugs. Accumulation in kidney (3.88%/g) at 1 h which reduces to 0.88%/g at 24 h post-injection indicates that some part of the radio-labelled drug is also eliminated from the body via renal route. Accumulation and retention in intestines were observed to be lower (Table 2) at all time points studied, associated probably with the weak absorption function of the radio-conjugate. The insignificant uptake of activity in the stomach (0.73%/g) is highly suggestive of its in vivo stability. The probable bone marrow suppression (myelotoxicity) will be monitored with increasing doses, dose-relating to the complex. Figure 4 presents the compartmental distribution of tissues between 1 and 24 h after injection.

Radio-conjugate was rapidly and randomly distributed into the brain tissue (0.12%/g for 1 h), but has disappeared quickly and reaches a minimum value in 24 h. Recently, some authors also reported that the MRI signal of the BBB quickly disappeared after transportation from the blood vessels to the brain (Figure 4(B)).[30,31]

**Figure 5.**
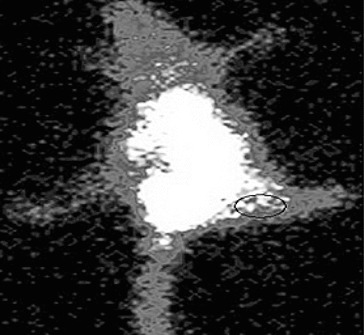
Whole body scintigraphic images of radio-labelled ^99m^Tc-SLENU nitrosourea in female BALB/c mice bearing s.c. EAT (in the right thigh) tumour implant.

Localization of ^99m^Tc-SLENU in Balb/c mice with EAT tumour at 2.5 h post-injection as seen by gamma camera imaging is presented in Figure 5.

Imaging was carried out at different time intervals. The mice depicted the beginning of accumulation of activity in tumour at 1 h, which reached the maximum at 2.5 h.

Other authors studied the nitroxides for clinical applications as well as to provide insight into the mechanisms of radiation cytotoxicity. The nitroxides have allowed us to explore the action mechanism of different chemotherapeutic agents for tumour imaging. Understanding these processes is important for the process of ameliorating the toxicity of therapies and to the rationale design of future agents.[32,33,34] Scintigram of EAT bearing mice at 2.5 h corroborated with biodistribution data. In C57BL/Lymphoma L1210 bearing mice the optimal dose for anticancer activity of SLENU was 45 mg/kg.[21,27,35] Maximum target-to-non-target ratio in Balb/c mice was obtained at 2.5 h which substantiates the potential of the nitroxides, represented as a new class for tumour scintigraph,[36] radioprotectors which may have application as general antioxidants and for in vivo radiotherapy.

## Conclusion

Nitroxides represent a new class of spin-labelled radioprotectors which also have application as general antioxidants. The stability and affinity towards the solid *Ehrlich ascites tumour* and other experimental indicate that these compounds have a potential for use in the in vivo visualization of tumours and probably can be applied for tumours such as modularly thyroid carcinoma, small cell lung cancer, ovarian cancer and several others. As a conclusion, these findings have led to the development of potential and selectiveness of nitroxyl. In additional toxicological studies and radiotherapy, the nitroxyl SLENU has promising applications for active brain-tumour targeting.
